# Approach to Abnormal Uterine Bleeding in Adolescents

**DOI:** 10.4274/jcrpe.galenos.2019.2019.S0200

**Published:** 2020-02-06

**Authors:** Cenk Yaşa, Funda Güngör Uğurlucan

**Affiliations:** 1 İstanbul University, İstanbul Faculty of Medicine, Department of Obstetrics and Gynecology, İstanbul, Turkey

**Keywords:** Abnormal uterine bleeding, adolescents, heavy menstrual bleeding, oral contraceptive pills, coagulopathy

## Abstract

This article reviews the current understanding and management of abnormal uterine bleeding (AUB) in adolescents. It is hoped that this review will provide readers with an approach to the evaluation and treatment of mild to severe uterine bleeding. AUB is a common problem which has significantly adverse effects on an affected adolescent’s quality of life. The most common underlying condition in AUB in adolescence is anovulation. During the evaluation, pregnancy, trauma and sexually transmitted diseases must be ruled out, regardless of history. It should be kept in mind that AUB during this period may be the first sign of underlying bleeding disorders. Although observation is sufficient in the mild form of AUB, at the other end of the spectrum life-threatening bleeding may necessitate the use of high doses of combined oral contraceptives, intravenous estrogen and/or interventional procedures.

## Introduction

Adolescents have frequent menstrual problems such as irregular menses, painful cycles and prolonged or heavy menstrual bleeding (HMB). Abnormal uterine bleeding (AUB) is defined as bleeding from uterine corpus that is abnormal in duration, volume, frequency and/or regularity. Due to immaturity of the hypothalamic-pituitary-ovarian (HPO) axis, AUB is common in adolescents (1,2). Furthermore, inherited or acquired bleeding diathesis may further intensify the existing hormonal imbalance and increase morbidity of the underlying condition. In addition to these problems, hyperprolactinemia, thyroid disorders and polycystic ovary syndrome (PCOS) are the common underlying endocrine disorders. AUB decreases quality of life, affects school attendance and limits sports and social activity participation (3). Although the management of this problem has evolved over time, the most important goal remains to alleviate the anxiety of both affected girls and their families and to identify the underlying medical conditions that may have chronic health effects for these girls. In this paper, the most common causes of AUB will be discussed and current management will be reviewed.

## Normal Menstrual Cycle in Adolescents and Classification of AUB

Although the age of onset of puberty has tended to decrease over the past few decades, the age of menarche has remained constant at 12-13 years (4). At present, more than 90% of girls are menstruating before age of 14 years. Menarche is generally considered as anovulatory bleeding. The time required for HPO axis maturation following menarche, which is thought to result in ovulatory cycles and subsequent regular bleeding, varies between six months and three years. Due to ovulatory dysfunction, in the following months after menarche, irregular and unpredictable, heavy and prolonged, and, rarely, skipped menses for less than three months may occur (5). Thus perception of “normal” menstrual cycle may vary in these girls and their families. As in adults, menstrual cycles are between 21 and 34 days, last for seven days or fewer, with an average blood loss of 30-40 mL leading to 3-6 pads or tampon usage per day (6). HMB is the most common form of AUB and is defined as excessive menstrual blood loss that interferes with a woman’s physical, social, emotional or material quality of life (7). Some additional signs of HMB include changing pad or tampon more often than every one to two hours, use of double hygiene protection, frequent soiling of clothes or bed sheets and blood clots more than one inch (2.54 cm) in diameter (8). The causes of HMB may be classified using the Polyp, Adenomyosis, Leiomyoma, Malignancy-Coagulopathy, Ovulatory dysfunction, Endometrial, Iatrogenic and Not yet classified (PALM-COEIN) classification which is divided into structural causes including PALM and hyperplasia and non-structural causes which include COEIN (9). The structural causes of HMB are rarely seen in the adolescent age group.

AUB might also be classified as acute or chronic. Acute AUB refers to an episode of heavy bleeding which is sufficient in quantity to require immediate intervention to prevent further blood loss. Abnormalities in quantity, regularity and/or timing in the last six months may all be defined as chronic AUB (10). Usually, chronic menstrual bleeding that exceeds 80 mL will result in anemia.

## Clinical Evaluation

AUB in adolescents is a challenging and often neglected problem. Cycle to cycle variability, differences in menstrual hygiene, the wide variety of menstrual hygiene pads or tampons available, inconsistency in giving information about menstrual regularity and bleeding amounts make initial assessment of AUB even more difficult in these girls (11). Patients and their families may not know what is “normal” and patients may not inform their families about menstrual irregularities. In addition, most bleeding disorders may not be as obvious until menarche. Although during this period, AUB may occur more frequently due to anovulation, bleeding disorders may also accompany this condition.

## Differential Diagnosis

In these girls, clinicians should evaluate the features of the menstrual cycle like a vital sign (12). An accurate history of patient’s cycles is the main issue for diagnosis, to determine if her experiences are normal or abnormal. The onset of menarche, cycle length, variability over time and the amount of menstrual bleeding should be evaluated. After providing a suitable conversation environment, sexual activity should be questioned. Pregnancy and its related complications should also be part of the initial investigation in girls presenting with AUB. Although AUB is often caused by anovulatory cycles, severe bleeding may be the first sign of an underlying condition and this may be a diagnosis of exclusion. PCOS, another cause of anovulatory cycles, should be kept in mind as a common underlying etiology of AUB, since it can easily be missed in this age group (13). Excessive bleeding during menarche can usually indicate an underlying bleeding disorder, while regular but excessive bleeding may also be indicative of bleeding disorders. Von Willebrand disease, platelet function defects, thrombocytopenia and clotting factor deficiencies are the most common bleeding disorders in adolescent girls that present with HMB. Up to 36% of adolescents with AUB may have an underlying coagulopathy (14). Using a screening tool for underlying bleeding diathesis in adolescents with AUB can assist the physician.

Initial screening for an underlying disorder of hemostasis in patients with excessive menstrual bleeding should be structured by the medical history. A positive screening result comprises the following circumstances:

- Heavy menstrual bleeding since menarche

- One of the following conditions:

- Postpartum hemorrhage,

- Surgery-related bleeding,

- Bleeding associated with dental work.

- Two or more of the following conditions:

- Bruising, one to two times per month,

- Epistaxis, one to two times per month,

- Frequent gum bleeding,

- Family history of bleeding symptoms.

Patients with a positive screening test should be considered for further evaluation, including consultation with a hematologist and testing for von Willebrand factor and ristocetin cofactor.

In intermenstrual bleeding, cervicitis and hormonal contraception may be implicated. In adolescents who do not respond to standard medical therapy, structural causes of bleeding should be excluded. Table 1 for common causes for AUB in adolescents.

## Physical Examination

When adolescents present with acute AUB, physical examination should focus on signs of acute blood loss and the etiology of bleeding. While tachycardia and orthostatic hypotension may be the only signs of severe anemia, it should be kept in mind that young patients will not present with clinical signs, despite severe anemia. While the presence of bruises and petechiae on the skin may indicate an underlying coagulation disorders, pallor may be seen due to anemia. In adolescents who are sexually active, trauma, foreign body, structural causes and pelvic inflammatory diseases can be investigated by pelvic and bimanual examination.

## Laboratory Evaluation and Imaging

Initial evaluation of adolescents presenting with acute AUB should include screening for pregnancy, anemia, bleeding disorders, iron deficiency and thyroid disease (15). Complete blood count, blood type, cross match and pregnancy test should be first line tests. In addition, partial thromboplastin time, prothrombin time, activated partial thromboplastin time and fibrinogen level are the initial evaluation for disorders of hemostasis. All adolescents with abnormal initial test or positive screening results for disorders of hemostasis should be evaluated by assessment of von Willebrand-ristocetin cofactor activity, von Willebrand antigen and factor VIII for diagnosis of von Willebrand disease and other coagulopathies (16).

Since exogenous estrogen use may increase von Willebrand Factor concentrations into the normal range, it is necessary to perform the test either before starting hormone treatment or seven days after the end of treatment to prevent false negative results (17). If patient’s history or physical examination findings are suggestive of PCOS, testosterone (free/total), DHEAS and prolactin should be evaluated. Sexually active adolescents should be screened for *Neisseria gonorhhea* and *Chylamidia trachomatis* infections with nucleic acid amplification tests.

Routine pelvic imaging is considered unnecessary since structural etiologies are rarely seen in this group. However, in the girls who do not respond to initial treatment, transabdominal ultrasonography may be more appropriate than transvaginal ultrasonography.

## Management

Most adolescents need outpatient management and reassurance that their menstrual cycles would become cyclic and ovulatory over time. However, treatment is required when AUB causes anemia or impairs quality of life (18). In these girls, the first line treatment is generally medical. Surgical options should be reserved for girls who can’t be managed by medical treatment. Acute AUB patients who are clinically unstable, have active bleeding or severe anemia should be hospitalized for management (19). A clinical decision should be made regarding intravenous crystalloid and blood or blood product transfusions, hormone treatment, and iron replacement, according to the severity of bleeding, clinical condition of the patient, hemodynamic stability and the underlying medical problem. If an underlying cause can be identified, appropriate specific treatment should be given.

## Management of Girls with Acute Bleeding

Girls with active, profuse, heavy bleeding (>1 pad per hour), presence of vital signs in conjunction with evidence of hypovolemia, orthostatic hypotension or hemoglobin (Hb) concentration <8 gr/dL due to bleeding are accepted as having severe AUB and should be hospitalized. If patients can tolerate oral intake, and in the absence of contraindications of estrogen treatment, monophasic combined oral contraceptive pills (OCP) containing 30-50 mcg ethinyl estradiol, can be used every 6-8 hours until bleeding diminishes, then taper to two and then one pill daily (19). If bleeding does not decrease after the first two doses of OCP or patients are not able to take oral hormone treatment, intravenous 25 mg conjugated estrogen every 4-6 hours should be considered until bleeding ceases (20). Most adolescents respond quickly to hormone treatment and iron supplementation and also tolerate anemia better than adults. Therefore, blood transfusion should be avoided as far as possible until the occurrence of hemodynamic instability or the presence of symptoms of severe anemia. There is no established Hb concentration for transfusion requirement. Furthermore, if transfusion is to be performed, one unit of packed red cell should be given and the need of further transfusion should be re-evaluated according to the subsequent status of the patient. Platelet transfusion is rarely required except in cases of severe thrombocytopenia or platelet disorder (21). In addition, when a deficiency of coagulation factors has been identified, clotting factors from plasma derived concentrate or recombinant agents might be needed (22). After cessation of bleeding, transition to maintenance treatment is required. High dose estrogen treatment can induce nausea and vomiting so anti-emetic agents should be begun in a prophylactic manner. If bleeding cannot be managed by these measures within 24-48 hours, consultation with a hematologist should be considered. During the maintenance period, continuous OCP (active pills only) which contain 30-50 mcg ethinyl estradiol with norgestrel or levonorgestrel (LNG), should be continued until Hb concentrations increase, or for longer in the presence of underlying bleeding disorders. In girls with a contraindication to estrogen-containing regimens, progesterone in the form of oral medroxyprogesterone acetate at a dose of 10-20 mg every 6-12 hours or oral norethindrone acetate at a dose of 5-10 mg every six hours are effective. Again, tapering of dose is begun after bleeding diminishes. Once the patient’s bleeding ceases and Hb level is stabilized, the patient could be discharged from hospital after toleration for oral therapy is established. During the maintenance period, oral iron supplementation, along with dietary counseling to increase iron intake, should be given until iron stores are restored as indicated by a normal ferritin concentration. An oral dose of iron of 60-120 mg per day is recommended. Recently, evidence has been presented which suggested that daily single dose use is better than multiple daily doses (23). If there are concerns about oral iron intake, intravenous iron treatment may be initiated for these girls during the hospitalization period. In girls who have menstrual bleeding under control, iron support is usually sufficient for 3-6 months. Complete blood count and iron studies should be performed to determine when to terminate the supplementation.

Although it is known that nonsteroidal anti-inflammatory drugs (NSAIDs) decrease menstrual bleeding in premenopausal women, NSAIDs should not be prescribed to these girls because this therapy may exacerbate AUB due to underlying bleeding disorders. Tranexamic acid is an anti-fibrinolytic agent that has been shown to be as effective in decreasing menstrual blood loss as OCP and improved the quality of life in adolescents (24). Concomitant use of tranexamic acid and OCP is contraindicated according to drug information because there is a hypothetical increased risk of thrombosis. However, the increased risk of thrombosis with combined use has not been demonstrated by long-term clinical experiences (25). Thus, despite this risk, OCP and tranexamic acid combination has been used in patients who fail to respond to treatment with OCP alone. The recommended dose of tranexamic acid is 1300 mg orally or 10 mg/kg intravenously (maximum 600 mg/dose) three times daily for up to five days (26). Aminocaproic acid, another anti-fibrinolytic agent, is both less effective and has more side effects (27). Desmopressin is a synthetic analogue of the vasopressin. It increases concentrations of von Willebrand Factor and Factor VIII. It also causes platelet adhesion. It is commonly used in type 1 von Willebrand disease, hemophilia and platelet function defects in the form of a nasal spray (28).

First-line medical management may fail to result in cessation of bleeding in some patients who may require interventional procedures and further evaluation. Even in cases of life-threatening bleeding, procedures such as uterine artery embolization, endometrial ablation and hysterectomy should not be performed, as these treatment modalities may cause future infertility. In these patients, pelvic ultrasonography and pelvic examination under general anesthesia might provide further evidence for clinical decision making. If the presence of clot or decidual cast is demonstrated by ultrasonography, uterine evacuation or suction curettage might be appropriate. An alternative intervention to stop bleeding may be intrauterine balloon insertion. Studies of balloon insertion, especially in women with postpartum bleeding, have reported efficacy in controlling bleeding. A Foley catheter is a low-risk, low-cost, and readily accessible intrauterine balloon to consider for young girls and adolescents (29). Since the uterine volume of these girls is small, the amount of inflation needed for effective tamponing is judged by the amount of wall resistance felt. The balloon may remain *in situ* for 12-24 hours while other treatments are given. After the bleeding stops, the balloon of the Foley catheter is gradually and carefully emptied and completely withdrawn. There is a risk of uterine perforation, endometrial damage and infection risk with this method (30). Prophylactic antibiotics should be given, as long as the balloon remains, for prevention of infection.

## Management of Girls with Mild or Moderate Bleeding

Girls with light or mild bleeding, indicated by normal Hb concentrations, should be reassured that observation is sufficient, unless there is an impairment of quality of life. NSAIDs can be used to reduce the amount of bleeding. If bleeding persists or becomes more severe, re-evaluation of the patient is required. If the Hb concentrations of these girls are found to be in the 10-12 gr/dL range, observation or OCP are valuable therapeutic options and 60 mg daily iron treatment should be commenced. If hormonal therapy is chosen, monophasic OCP, with 30-50 mcg ethinyl estradiol content, can be used every 8-12 hours until bleeding slows, then the therapy should be tapered to one pill daily over the course of a few days and therapy should be continued for at lesat 21 days.

In the presence of moderate bleeding or Hb concentration in the range 8-10 gr/dL, oral contraceptive treatment should be initiated as described above and continued until the Hb concentration is above 12 gr/dL with at least six months of iron supplementation. In the presence of a contraindication to estrogen therapy or alternative treatment in adolescents with anemia, progesterone therapy can be an option. Available progesterone therapies are oral medroxyprogesterone acetate (10 mg/day), micronized oral progesterone (200 mg/day) or norethindrone acetate (2.5-5 mg/day), which should be given for 12 days in every cycle (31).

## Long-term Management of Girls with Bleeding Disorders

After acute menstrual bleeding ceases, these girls require treatment for long-term bleeding control. In addition to diet optimization and iron supplementation, hormonal treatments are used. Hormonal treatments include OCP, oral, injectable and implantable progesterone and the LNG-releasing intrauterine device (LNG-IUD). For OCP, continuous or extended-cycle regimes are recommended for stabilization of the endometrium. Combinations of 30-50 mcg of ethinyl estradiol and levonorgestrel or norgestrel are more effective in reducing bleeding than in low-dose and new generation progesterone-containing preparations. Depot medroxyprogesterone acetate is also used for long-term bleeding control. Since there is a risk of hematoma with intramuscular administration, subcutaneous injection is recommended. To reduce the likelihood of initial breakthrough bleeding, therapy is applied more frequently than the usual 12-week cycle (32). Since daily, monthly and quarterly use of some formulas can be difficult for adolescents the LNG-IUD may be preferred. The LNG-IUD is active for up to five years after being placed in the uterine cavity. Additional benefits of the LNG-IUD are highly effective contraception, higher continuation rates and higher satisfaction rates when used for bleeding control compared with OCP in an adolescent population (33). In adolescents with bleeding disorders, the LNG-IUD has been demonstrated to be effective in controlling menstrual bleeding (34,35). Since breakthrough bleeding caused by etonorgestrel implants is a common side effect, it is not usually used for the treatment of AUB.

## Conclusion

AUB in adolescents may be acceptable at the beginning of the reproductive age when menstrual cycle regularity is not established, or may be the first sign of a severe underlying bleeding disorder. Girls with AUB should be evaluated with care and a wide differential diagnosis should be borne in mind. Medical therapy is usually an effective and sufficient treatment. Generally, adolescents respond well to therapy. Hematology consultation, imaging methods and clinical intervention should be considered in patients who do not respond to treatment.

## Figures and Tables

**Table 1:**
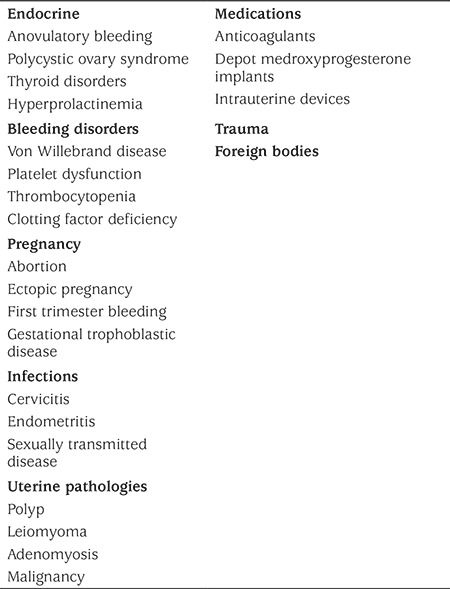
Differential diagnosis of abnormal uterine bleeding in adolescents
